# [Retracted] Scutellarein suppresses Aβ-induced memory impairment via inhibition of the NF-κB pathway *in vivo* and *in vitro*

**DOI:** 10.3892/ol.2025.15403

**Published:** 2025-11-26

**Authors:** Xiao-Wei Huang, Yan Xu, Xin Sui, He Lin, Jia-Ming Xu, Dong Han, Dou-Dan Ye, Guang-Fu Lv, Yue-Xin Liu, Xiao-Bo Qu, Ming-Hua Duan

Oncol Lett 17: 5581–5589, 2019; DOI: 10.3892/ol.2019.10274

Following the publication of the above paper, it was drawn to the Editor's attention by a concerned reader that both the immunofluoresecence data and the western blot data shown in Fig. 3 on p. 5585 had already appeared in an article written by different authors at different research institutes that was published previously in the journal *International Journal of Molecular Medicine*.

Owing to the fact that the contentious data in the above article had already been published prior to its submission to *Oncology Letters*, the Editor has decided that this paper should be retracted from the Journal. The authors were asked for an explanation to account for these concerns, but the Editorial Office did not receive a reply. The Editor apologizes to the readership for any inconvenience caused.

